# Mayenite Synthesis from Hydroxide Precursors: Structure Formation and Active Sites on Its Surface

**DOI:** 10.3390/ma15030778

**Published:** 2022-01-20

**Authors:** Aleksandr V. Kapishnikov, Roman M. Kenzhin, Anton P. Koskin, Alexander M. Volodin, Pavel V. Geydt

**Affiliations:** 1 Laboratory of Functional Diagnostics of Low-Dimensional Structures for Nanoelectronics, Department of Physics, Novosibirsk State University, Pirogova Str., 2, 630090 Novosibirsk, Russia; r.kenzhin@nsu.ru; 2 Federal Research Center Boreskov Institute of Catalysis, Siberian Branch of the Russian Academy of Sciences, Prospekt Lavrentieva, 5, 630090 Novosibirsk, Russia; koskin@catalysis.ru (A.P.K.); volodin@catalysis.ru (A.M.V.)

**Keywords:** mayenite, XRD, catalytic materials, spin probe method, surface active sites

## Abstract

We studied the formation process of a mayenite structure from hydroxide precursors in different gas media. According to X-ray diffraction data, this method allows a well-crystallized mayenite (Ca_12_Al_14_O_33_ or C12A7) phase to be obtained at low (500–900 °C) temperatures with an insignificant impurity of CaO. It was shown that the lattice parameters for C12A7 obtained in an inert atmosphere (Ar) were lower when compared with similar samples in the air. These results can be explained by the different levels of oxygen nonstoichiometry in the resulting phase. We noted that sintering and crystallization of mayenite proceeds at lower temperatures in Ar than in the air medium. We found the presence of donor and acceptor active sites on the surface of mayenite, which was detected by the spin probe method. The specific (per unit surface) concentration of such sites (2.5 × 10^16^ m^−2^ and 1.5 × 10^15^ m^−2^ for donor and acceptor sites, respectively) is comparable to that of γ-Al_2_O_3_, which is traditionally used as catalyst support. This allows it to be used in adsorption and catalytic technologies, taking into account its high specific surface area (~30–50 m^2^/g at a low synthesis temperature).

## 1. Introduction

Recently, a significant number of works were dedicated to the study of the properties of calcium aluminates with a mayenite structure (Ca_12_Al_14_O_33_ or 12CaO·7Al_2_O_3_, also abbreviated as C12A7). This is due to the possible wide range of variations in the functional properties of this material, caused by changing the composition of its anionic sublattice, which occurs with the retention of its cationic framework. The unique chemical and electrophysical properties of these materials were discovered almost two decades ago [1,2] and later studied in detail in numerous works of the H. Hosono group [3,4,5,6,7,8,9,10]. These compounds contain a stable cationic framework [Ca_24_Al_28_O_64_]^4+^ and a sufficiently mobile anionic sublattice 4X^−^. The chemical and electrical properties of the obtained materials can be varied over a very wide range by simply replacing the X^−^ anions. The composition of the unit cell of such a compound can be described by the formula:

1 unit cell = [Ca_24_Al_28_O_64_]^4+^ • 4X^−^,

where X^−^ = H^−^, O^2−^, O^−^ , O_2_^−^, OH^−^, Cl^−^, F^−^, e^−^.

The materials containing unstable (active) forms of anions, e.g., H^−^, O^−^, O_2_^−^, and especially, e^−^, are prominent in this series. Thus, X^−^ = O^−^ can act as suppliers for materials with reactive radical O^−^ anions for chemical and catalytic oxidation reactions [5,11,12], while electrides (X^−^ = e^−^ [2]) can be used in emission devices [13,14,15] and memory elements [16,17], acting as electron donors in organic synthesis reactions [18], or as electron donor support materials for heterogeneous catalysts based on noble metals [7,9,10]. A wide range of possible applications has stimulated the development of synthesis methods focused on the fabrication of these materials in desired forms (1D, 2D, 3D) with the required functional properties. Particularly, the key parameters for the use of mayenite in catalytic and adsorption technologies are the specific surface area (SSA) of the material and the presence of various types of active sites on its surface. At the same time, emission devices require a low work function for the ceramic composite materials that can be used in them. Whereas the ReRAM memory elements require a significant difference in concentration of carriers; they are mobile and have a high cyclic stability of the crystal lattice for active layer materials.

Previously, we proposed a simple method for the synthesis of pure-phase mayenite with a sufficiently high SSA by sintering of the hydroxide precursors [19,20]. A similar approach to the synthesis of mayenite was used in a recently published work by another research group [21]. It should be noted that acceptor and donor sites on the surface of oxide systems studied using the spin probe method play an important role in many catalytic reactions on acid catalysts [22,23], and are also responsible for the stabilization of active forms of supported noble metals [24,25,26,27]. In this regard, the study of such sites for mayenite samples synthesized under various conditions is of particular interest. Despite the fairly widespread use of inorganic electrides with a mayenite structure as effective support materials for catalysts [7,9,11], a detailed characterization of the active sites of mayenite (e.g., their types and concentrations) has not yet been reported in the literature. The aim of the present work was to conduct a comparative study of the evolution of the C12A7 structure and active sites on its surface during the calcination of this material in a wide temperature range and different gas atmosphere.

## 2. Materials and Methods

The synthesis of mayenite was carried out using a method similar that described in works [19,20]. The starting materials for the synthesis were aluminum hydroxide (Pural SB-1, pseudo-boehmite, >99.9%, Condea, Brunsbüttel, Germany) and calcium carbonate (extra purity, >99.97%, Reachim, Penza, Russia). The first stage of the synthesis was the preparation of CaO by decomposing CaCO_3_ in a muffle in the air at 950 °C for 6 h. Next, CaO was added to a suspension of aluminum hydroxide (100 g/L) in distilled water at room temperature and continuously stirred. The final component ratio corresponded to the stoichiometry of mayenite (12CaO·7Al_2_O_3_). The mixture was thoroughly stirred in distilled water for 10 h, filtered, and dried at 110 °C. Then, it was calcined in a muffle furnace in the air at 250 °C for 6 h. The obtained C12A7-250 sample was used as a starting material for further synthesis. Then, the obtained samples were calcined in the air and an Ar flow (16.67 sccm). The initial sample C12A7-250 was heated in all cases at a rate of 3 °C/min. The temperature was maintained for 6 h, and then the sample was cooled down to room temperature at the same rate of 3 °C/min. The samples obtained by calcining in the air are hereinafter referred to as C12A7-T, where T is the calcination temperature. The designation C12A7-T-Ar will be used for the samples calcined in Ar atmosphere. 

The phase composition of the samples was studied by X-ray powder diffraction on an ARL X’tra (ThermoFisher, Ecublens, Switzerland) diffractometer with Cu Kα radiation (the X-ray wavelength λ = 1.5418 Å). The data were recorded in the 2θ range 15–50° with a resolution of the Bragg reflection angle 2θ = 0.02°. The calculation and refinement of the unit cell parameters were carried out according to the positions of 3–4 separate peaks by the least-squares method using the POLYCRYSTAL software package [28]. An approximation of the reflections was conducted using the Cauchy–Lorentz functions. The coherent scattering region (CSR or crystallite) sizes D were determined according to the Selyakov–Scherrer formula: D = 0.9λ/(β·cosθ), where β is the broadening of diffraction reflection peaks. The semi-quantitative estimation of the fractions of the crystalline and nanocrystalline (dispersive) components of mayenite was based on an integral intensity analysis, similar to the method described in [29,30]. After subtracting the background intensity, the peaks of mayenite were analyzed as a superposition of two reflections from the crystalline and dispersive phases. The fraction of the crystalline component Wcr was estimated by the formula: Wcr = Icr/(Icr + In), where Icr and In are integral intensities of the diffraction peaks of the crystalline and dispersive phases, respectively.

The concentration of various types of active sites on the sample surface was experimentally determined using spin probe methods. The probe adsorption and spectrum recording procedures were described in detail in our earlier publications [22,24,31]. The Electron Paramagnetic Resonance (EPR) spectra were recorded at room temperature using an ERS-221 spectrometer (Center of Scientific Instruments Engineering, Leipzig, German Democratic Republic) operating in the X-band. The concentrations of the paramagnetic species were determined by numerical double integration with baseline compensation. A dark-colored, crystalline powder 2,2-diphenyl-1-picrylhydrazyl (DPPH) primary standard for quantitative EPR spectrometry was used for calibration of the spectrometer prior to the study. To determine the concentration of donor sites on the surface of the studied systems, we used radical anions emerging during the adsorption of acceptor molecules, such as 1,3,5-trinitrobenzene (TNB) spin probes [24,31]. To determine the concentration of acceptor (acid) sites, we used the radicals emerging during the adsorption of anthracene [22]. Before the adsorption of the probe molecules, the samples were activated in the air at 500 °C for 3 h.

Specific surface areas were determined on the basis of the Brunauer–Emmett–Teller (BET) theory. The data for this method were obtained by low-temperature argon adsorption using an Accelerated Surface Area and Porosimetry system ASAP-2400 (Micromeritics Instrument Corp., Norcross, GA, USA).

## 3. Results

### 3.1. X-ray Diffraction Analysis of the Evolution of C12A7 Samples after Heating in Air and An Ar Flow

**Figure 1:**
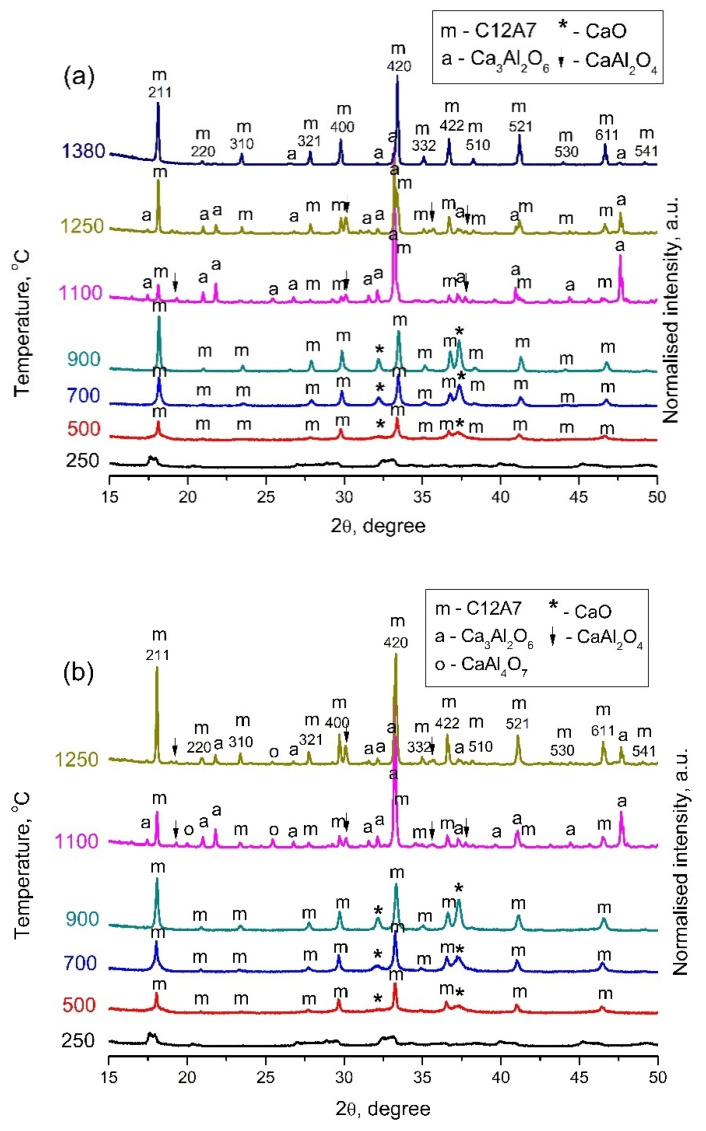
Diffraction patterns of samples obtained in inert Ar atmosphere (**a**) and in air (**b**) at different temperatures.

**Figure 2:**
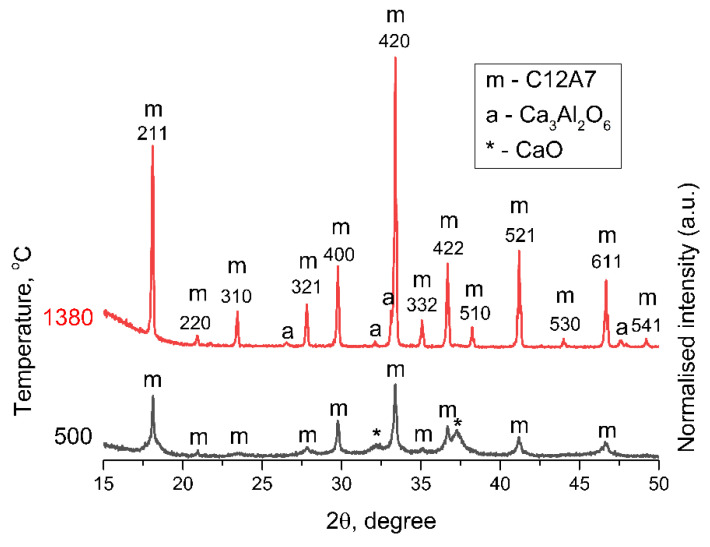
Comparison of diffraction patterns for samples obtained at 500 °C and 1380 °C in Ar atmosphere.

According to the X-ray phase analysis, the initial sample C12A7-250 appeared to be a mixture of amorphous calcium and aluminum hydroxides. Due to the difficulty in determining the positions of the peaks, it can only be argued that the sample contained the Ca_3_Al_2_(OH)_12_, Ca(OH)_2_, and AlO(OH) phases. The samples obtained upon further calcination in Ar and air contained several impurities with the primary Ca_12_Al_14_O_33_ phase (JCPDS № 9-413). The formation of mayenite in Ar (Figure 1a) and air (Figure 1b) proceeded in a similar way. Traces of CaO (JCPDS № 37-1497) were also present in the phase composition of samples, apart from the C12A7 at synthesis temperatures ranging from 500 °C to 900 °C. A significant broadening is also observed at the bottom of the reflections from the mayenite phase (Figure 2). This effect can be caused by a bimodal distribution of the particle size and associated with the contribution from nanocrystalline mayenite with a CSR of about 10 nm. The partial decomposition of C12A7 to the Ca_3_Al_2_O_6_ (C3A, JCPDS № 38-1429) and CaAl_2_O_4_ (CA, JCPDS № 23-1036) phases occurred at 1100 °C in both atmospheres, according to Figure 1. This decomposition process, similar for both media, was mentioned in previous work by Eufinger et al. [32]. An insignificant difference was observed, i.e., the admixture of the CaAl_4_O_7_ (CA2, JCPDS № 23-1037) phase appeared in the phase composition during the heating above 900 °C in air (Figure 1b). Remarkably, when the temperature increased to 1250 °C, calcium aluminate phases reacted and formed the C12A7 phase again. At 1380 °C in Ar, the formation of well-crystallized mayenite with a small admixture of C3A was observed.

The comparison of the initial (500 °C) and final (1380 °C) states of the material is specially presented in Figure 2 to designate the phase purity of the heated material after two distinct heating conditions.

The structural state of C12A7 appeared to be different for the samples synthesized in air and in Ar. Table 1 presents the calculated C12A7 lattice parameters for different synthesis conditions. For mayenite obtained in an Ar medium at low temperatures (500–900 °C), our experimental values were close to the values for C12A7:O^2−^ that were previously reported by other groups [33,34]. The increased value of the lattice parameter at 500 °C can be associated with a high proportion of the C12A7:OH^−^ states in the structure. When the temperature rises to 700–900 °C, the dehydration and substitution of OH^−^ groups by O^2−^ anions occurs, which can reduce the lattice parameter. The increased value of the lattice parameter at 1100 °C can be associated with the decomposition reaction of mayenite at this temperature, which can affect the state of its unit cell. With a further increase in temperature, the lattice parameter becomes close to the value for electron-injected C12A7:e^−^ that was mentioned in previous works [35,36]. Remarkably, in the case of heating in the air, the values of the lattice parameters were higher than in the Ar medium. A similar value for the parameter of 12.070 Å was mentioned in [37], even though it was acquired by the theoretical DFT method. The nature of the change in the parameters with increasing temperature is similar to calcination in Ar, except that the radical states of oxygen-like O^−^ and O_2_^−^ are formed in the framework in an oxygen-containing medium [5,38]. In [39], the values of possible lattice parameters were shown depending on oxygen stoichiometry. In accordance with “ab initio” calculations, the formation of the superstoichiometric state in Ca_12_Al_14_O_32+δ_ is possible in the air at 25–750 °C [39]. Our experimental dataset (Table 1) can be associated with the lattice parameters for the stoichiometric parameter δ in the range of 4–6 (a = 12.02−12.04 Å). This provides evidence for the dominance of O_2_^−^ clathrate forms in the mayenite structure obtained in the air medium.

The genesis of the microstructure during both heating in the air and an Ar atmosphere is similar, with minor differences (see Table 2). Calcination at 500 °C resulted in obtaining quietly dispersive systems with high SSA values. The increasing synthesis temperature facilitated the sintering of nanocrystalline mayenite and decreased SSA in the temperature range of 500–900 °C for both Ar and air media. This was also verified by the value of the fraction of the dispersive phase, which sharply decreased for the corresponding temperatures in both gas media. This sintering process was accompanied by lowering the CSR values, which can be explained by the formation of new, well-crystallized, small particles from the dispersive fraction. According to the data presented in Table 2, the sintering of nanocrystalline mayenite particles occurs in an Ar atmosphere at a lower temperature than in the air. A subsequent increase in calcination temperature promotes the formation of a well-crystallized mayenite phase. The decomposition process at 1100 °C does not significantly affect crystallite sizes. Most likely, this process occurs in smaller C12A7 particles while the larger particles persist.

### 3.2. Evolution of Active Sites on the Surface of C12A7 Samples after Heating in Air and an Ar Flow

Active sites on the surface of solids are caused by the presence of various types of coordinatively unsaturated structures. They significantly determine the result of the interaction of the surface with molecules of the external medium in gas or liquid phases. The most important role is played by active sites when using materials in catalytic and adsorption technologies. Spin probe methods based on the use of EPR spectroscopy detects and determines the concentration of various types of active sites on the surface of dispersed oxide systems with acidic and basic properties [22,23,24,25,31]. Thus, effective spin probes for the study of catalysts with basic properties are radical anions of aromatic nitro compounds emerging during their adsorption on donor surface sites [24,25,31], while radical cations emerge from the adsorption of donor molecules of aromatic compounds on the acceptor sites of the surface [22,23]. In the final part of the present work, we studied such sites on the surface of C12A7 samples previously characterized by the XRD method. We used γ-Al_2_O_3_ and CaO, individual components that are often used as precursors for the synthesis of mayenite, as reference samples in these experiments.

**Figure 3:**
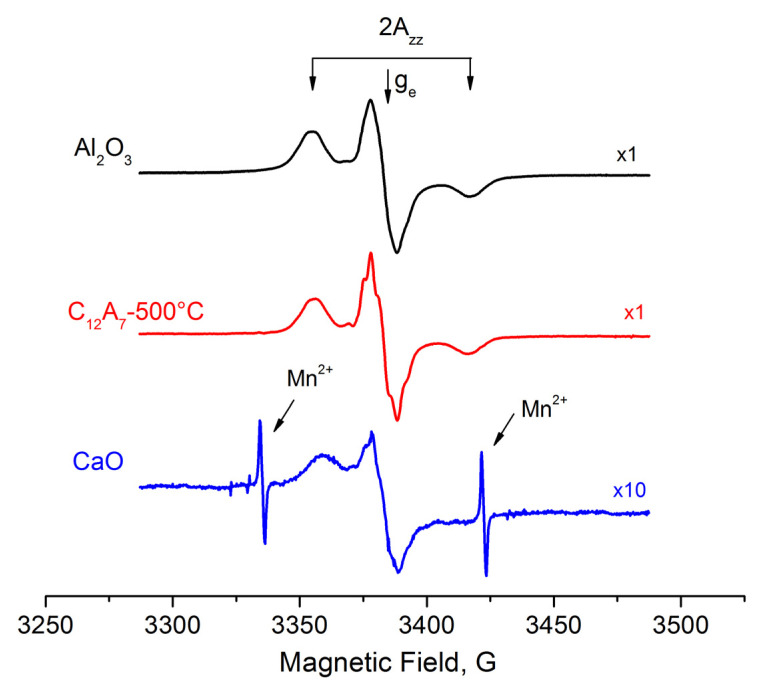
EPR spectra of radical anions on the surface of γ-Al_2_O_3_, C12A7-500, and CaO samples activated at 500 °C, appearing after the adsorption of 1,3,5-trinitrobenzene.

Figure 3 shows the EPR spectra emerging from the adsorption of 1,3,5-trinitrobenzene (TNB) molecules on the donor sites of the surface of the studied samples. These are the spectra of TNB radical anions stabilized at donor surface sites [24,25,31]. They represent a three-component signal due to a hyperfine interaction with nitrogen atoms. The sites responsible for the formation of these radical anions are present on mayenite, and their spectra are similar to those observed for γ-Al_2_O_3_. Slightly different hyperfine coupling Azz values (31 G for γ-Al_2_O_3_, 30 G for C12A7 and 25 G for CaO) for these samples correlate well, with an increase in the strength of the main sites in this series.

**Figure 4:**
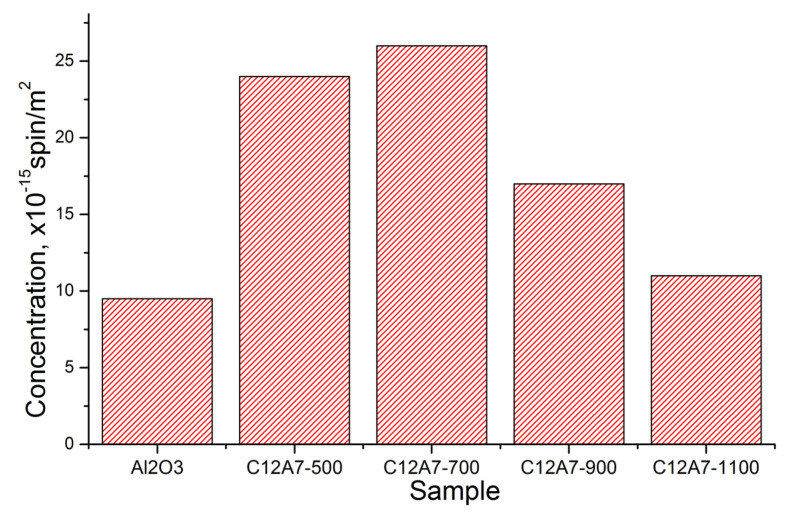
The concentration of TNB-radical anions per unit surface of the samples.

Donor sites on the surface of oxide materials play an important role in the stabilization of the deposited noble metals [24,25,26,27] and determine the activity of such materials in catalytic oxidation reactions. In this regard, it was of interest to compare the concentrations of the donor sites detected by the spin probe method for C12A7-T materials and γ-Al_2_O_3_, i.e., a traditional support for various catalysts. Figure 4 shows the results of this comparison. As can be seen from the data presented in Table 2, the SSA values for these samples are different. For this reason, the data in Figure 4 are referred to as the unit area of the samples.

It is observed that the heating of C12A7 samples in the temperature range from 500 °C to 700 °C has practically no effect on the specific surface concentration of the detected donor sites and is more than twice that of γ-Al_2_O_3_. This decrease, together with an increase in temperature up to 1100 °C may be due to the reversible decomposition of the mayenite phase and the formation of C3A and CA phases (See Figure 1) with other surface properties.

The presence and strength of the electron-acceptor sites on the surface of oxide systems are associated with their acidic properties. In many cases, their concentration can determine the efficiency of the catalytic reactions due to the acidic properties of the catalyst [22,23]. In the present work, we used radical cations emerging after the adsorption of anthracene as spin probes to estimate the concentration of the acceptor sites.

**Figure 5:**
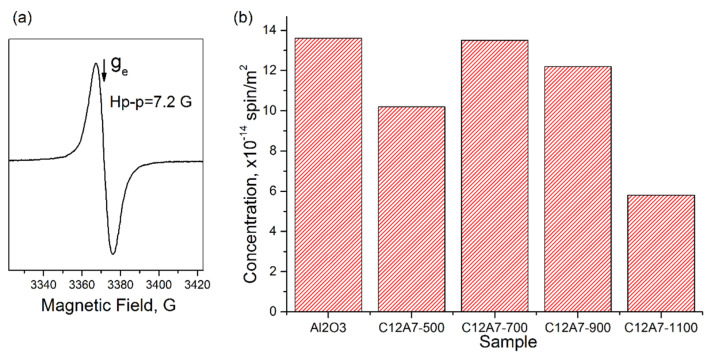
(**a**) Typical EPR spectrum of radicals appearing after the adsorption of anthracene on γ-Al_2_O_3_ and C12A7 samples. (**b**) The concentration of radicals arising from the adsorption of anthracene molecules on acceptor sites. The given concentration values are referred to as the unit surface of the samples.

Figure 5a shows a typical EPR spectrum that appears after the adsorption of anthracene molecules on the studied samples. It is a singlet line (g = 2.0036, Hp-p = 7.2 G), which appeared to be the same for the γ-Al_2_O_3_ and C12A7 samples. However, the concentration of these radicals differs, which was determined by the concentration of acceptor sites on the surface of the studied materials. For CaO, the formation of these radicals was not observed due to the lack of acidic properties.

Figure 5b shows a comparison of the specific (per unit surface) concentration of these radicals for the studied samples. It is clearly seen that the concentration of acceptor sites on the surface of the C12A7 and γ-Al_2_O_3_ samples are similar up to a calcination temperature of 900 °C. This decrease in concentration for samples calcined at 1100 °C, as in the case of TNB radical anions, is most likely related to a decrease in the fraction of the mayenite phase in the studied material due to its decomposition.

From the presented results, it can be concluded that the specific concentrations of acceptor sites on the surface of all studied C12A7 samples are similar to those of γ-Al_2_O_3_. At the same time, the specific concentration of donor sites on mayenite is almost twice as high as γ-Al_2_O_3_. Mayenite is most likely more effective as a support for catalysts in comparison with γ-Al_2_O_3_, for which the presence of donor sites plays an important role. Specifically, these donor sites can consist of noble metal oxidation catalysts [24,25,26,27].

## 4. Conclusions

Solid-state transformations of hydroxide precursors Ca(OH)_2_ and Al(OH)_3_, followed by the controlled heating of the obtained mayenite ceramic material in different gas media, was studied in this work. The XRD analysis showed that this method allows predominantly C12A7 to be obtained, but it also contains some admixtures such as CaO for low temperatures (500–900 °C) or C3A, CA, and CA2 for high (1100–1380 °C) temperatures. The lattice parameters for mayenite obtained in Ar are lower than for similar samples in the air. Our results were caused by different levels of oxygen non-stoichiometry in the obtained mayenite. The sintering and crystallization of mayenite in Ar proceeds at lower temperatures than in the air medium. We detected the donor and acceptor active sites on the surface of mayenite by the spin probe method. The specific (per unit surface) concentration of such sites is comparable to that of γ-Al_2_O_3_, which is traditionally used as a catalyst support. Thus, we highlight the capability of using mayenite in adsorption and catalytic technologies, taking into account its high specific surface area ~30–50 m^2^/g already at a low synthesis temperature. The obtained phases can be used in various deposition techniques to produce various desired structures, which are proposed for further applications in catalysis, ceramics, memory elements and electron-emitting devices.

## Figures and Tables

**Table 1:** Lattice parameters for C12A7 synthesized at different temperatures in Ar and air media.

T, °C	a, Å		Ref.
	Ar	Air	
500	11.995 ± 0.003	12.041 ± 0.007	This work
700	11.981 ± 0.003	12.038 ± 0.004	This work
900	11.982 ± 0.002	12.022 ± 0.001	This work
1100	11.998 ± 0.003	12.025 ± 0.003	This work
1250	11.998 ± 0.003	12.033 ± 0.004	This work
1380	11.999 ± 0.001	-	This work
1200	-	11.989	[33]
1350	-	11.979	[34]
1300	11.993	-	[35]
1300	11.999	-	[36]

**Table 2:** Crystallite sizes of crystalline C12A7 (D), fractions of crystalline (Wcr) and nanocrystalline (Wn) C12A7, and SSA for samples obtained in Ar and air at different temperatures.

T, °C	D, nm		Wcr, %		Wn, %		SSA, m^2^/g	
	Ar	Air	Ar	Air	Ar	Air	Ar	Air
500	96	77	23	20	77	80	61	63
700	53	71	65	53	35	47	54	45
900	70	57	-	70	-	30	26	38
1100	92	85	-		-		14	12
1250	100	90	-		-		1	2

## Data Availability

The data presented in this study are available on request from the corresponding authors.
